# Synthesis of the C_3_ and C_1_ Constitutional Isomers of Trifluorosubphthalocyanine and Their Fluorescence within MDA-MB-231 Breast Tumor Cells

**DOI:** 10.3390/molecules24213832

**Published:** 2019-10-24

**Authors:** Rosemarie L. Calandrino, Katherine J. McAuliffe, Lauren E. Dolmage, Evan R. Trivedi

**Affiliations:** Department of Chemistry, Oakland University, Rochester, MI 48309, USA; rcalandrino@oakland.edu (R.L.C.); katherine.mcauliffe@duke.edu (K.J.M.); laurendolmage@oakland.edu (L.E.D.)

**Keywords:** fluorescent probes, MDA-MB-231, subphthalocyanine

## Abstract

Metal tetrapyrrole macrocycles such as porphyrins and chlorins are ubiquitous in nature. Synthetic analogs, including phthalocyanines, have found applications in medicine, particularly as photosensitizers for photodynamic therapy and as fluorescent imaging probes. Tripyrrolic macrocycles, called subphthalocyanines (SPcs) with a smaller boron atom at their core, have similar potential as optical agents. We have recently reported a series of mixed fluorinated SPcs with varying aromaticity, showing that electronic absorption and emission are synthetically tunable across the far visible region, and that the inclusion of 4–12 peripheral fluorine atoms results in strong fluorescence within MDA-MB-231 breast tumor cells. Further probing this system, we report herein the synthesis and characterization of boron trifluorosubphthalocyanine chloride (F_3_SPc). The constitutional isomers F_3_SPc(C_3_) and F_3_SPc(C_1_) are readily separable by chromatography, and their identity and purity have been confirmed by ^1^H NMR, ^19^F NMR, HR APCI-MS, and HPLC. Unsurprisingly, these structurally similar F_3_SPcs have identical electronic absorption (λ_max_ = 557 nm; tetrahydrofuran (THF)) and emission (λ_em_ = 574 nm; Φ_f_ = 0.27–0.28; THF). Strong fluorescence from MDA-MB-231 breast tumor cells was observed following treatment with F_3_SPc(C_3_) and F_3_SPc(C_1_) (50 µM F_3_SPc, 15 min), further highlighting the importance of even a limited number of peripheral fluorine atoms for this type of application.

## 1. Introduction

Subphthalocyanines (SPcs), contracted tripyrrolic cousins of tetrapyrrole phthalocyanines (Pcs), were first prepared in 1972 as a templated cyclization of phthalonitrile derivatives around a central boron atom [1]. There is a rich chemistry surrounding these optically active boron macrocycles, and their structural diversity and applicability have been reviewed extensively [2,3]. More recently, beryllium subphthalocyanines have emerged as equally promising molecules [4]; however, toxicity issues have thus far precluded the widespread attention that boron subphthalocyanines have garnered. Notably relevant to this communication, halogenated SPcs have been used in photovoltaic and organic light–emitting diode technologies [5,6,7,8], and other SPcs have been applied in biological systems as anti-cancer agents [9], optical imaging agents [10], and as antibacterial agents [11]. We intend to build upon these limited biological studies by creating a library of fluorinated SPcs for application as either photodynamic therapeutics or fluorescent probes. These applications are well developed for the tetrapyrrole porphyrins and phthalocyanines [12,13,14,15], but understudied for the SPc family. Our choice of fluorinated SPcs for these purposes is two-fold: Peripheral electron-withdrawing groups allow for varied post-cyclization reactivity [3], and the C–F bond imparts greater pharmacokinetic and physiochemical stability in 20% of commercial pharmaceuticals [16,17,18,19].

We have recently studied a series of mixed fluorinated SPcs and subnapthalocyanines (SnPcs), made through co-cyclization of tetrafluorophthalonitrile, and phthalonitrile (SPcs) or 2,3-napthalenedicarbonitrile (SnPcs) [20]. These two reactions resulted in a series of macrocycles with a variable number of peripheral fluorine atoms (#F = 0, 4, 8, 12) and varied aromaticity (π e^−^ = 14, 16, 18, 20). These studies showed that electronic absorption and emission profiles are highly tunable across the visible region through variation of peripheral F-atoms and aromaticity. Subsequently, it was shown that further modulation and tunability of photophysical properties could be achieved through inclusion of peripheral electron-donating thioethers [21] or fused thiadazole rings [22]. When applied as fluorescent imaging probes in vitro in MDA-MB-231 breast tumor cells, the presence of peripheral fluorine proved to be imperative; there was a marked increase in intracellular fluorescence from subphthalocyanine (#F = 0) to tetrafluorosubphthalocyanine (#F = 4). Considering that these two molecules have nearly identical fluorescence brightness (ε · Φ_f_), we attributed the increase in fluorescence to enhanced cellular uptake, presumably due to the F-atoms’ modulation of polarity or lipophilicity. Other pyrrolic macrocycles with peripheral C–F bonds are known to undergo S_N_Ar reactivity, thereby replacing peripheral F-atoms with a nucleophile [23], and there is extensive evidence of axial reactivity across the B–Cl bond [24]. The possibility therefore exists that fluorinated SPcs undergo similar reactivity in the biological milieu.

**Figure 1:**
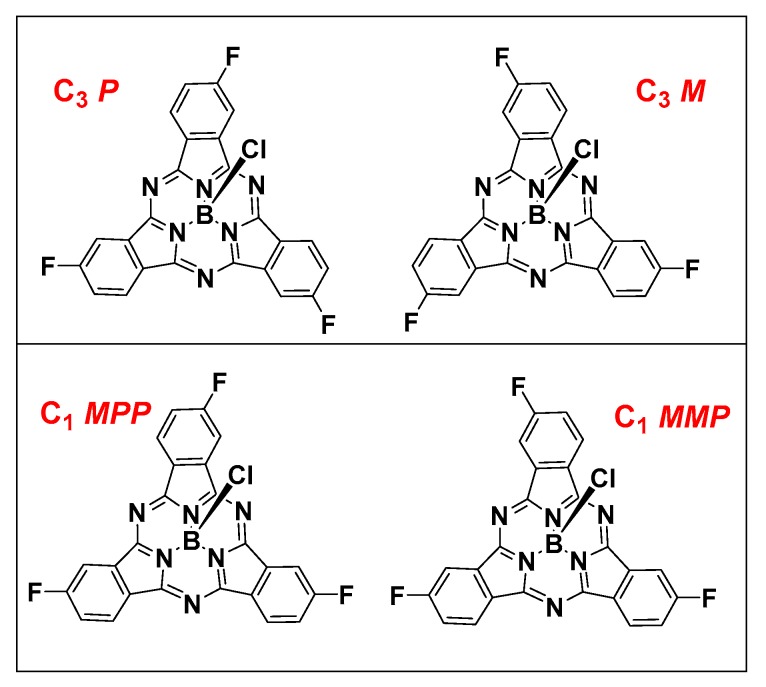
C_3_ constitutional isomers (**upper**) and C_1_ constitutional isomers (**lower**) of F_3_SPcs.

The mono-cyclization of a C_2v_ phthalonitrile with BCl_3_ results in a single C_3v_ SPc product. However, cyclization with a non-C_2v_ phthalonitrile results in a mixture of two constitutional isomers, statistically forming isomers in a 1:3 C_3_ to C_1_ ratio (Figure 1). These constitutional isomers are further subdivided into a racemic mixture of enantiomers due to the inherent axial chirality of non-C_3v_ symmetric SPcs. Despite the similar structure of constitutional isomers, there are examples in the literature where they can be separated chromatographically [25], and enantiomers can be further resolved by chiral HPLC [26].

Owing to their nearly identical photophysical properties, constitutional isomers are used in many practical applications as a mixture. Indeed, the trifluorinated SPc discussed herein has been applied in photovoltaics as a mixture of constitutional isomers [8], and considered as a mixture computationally [27]. Use of the mixture with this platform for our future purposes—to study peripheral reactivity of the C–F bond—would be difficult at best. We report herein, the successful isolation of the C_3_ and C_1_ isomers of F_3_SPc, and associated photophysical properties relevant to their application as fluorescent imaging probes in MDA-MB-231 breast tumor cells.

## 2. Results and Discussion

### 2.1. Synthesis and Characterization of C_3_ and C_1_ Constitutional Isomers of Trifluorosubphthalcyanine

**Scheme 1:**
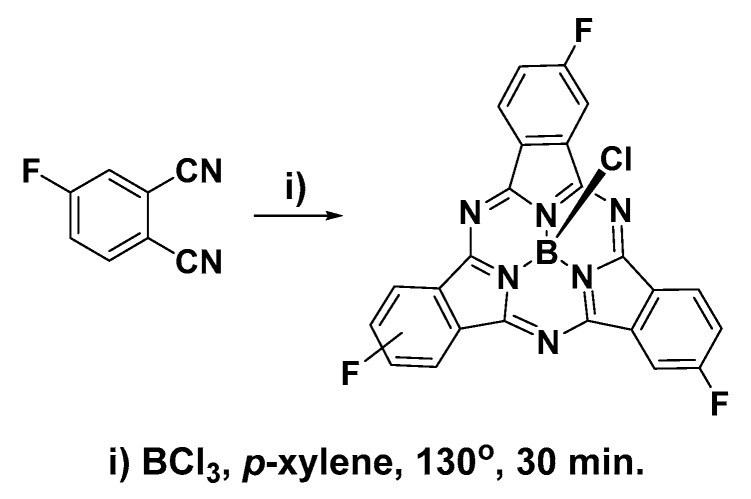
Synthesis of trifluorosubphthalocyanines.

Cyclization of 4-fluorophthalonitrile in the presence of BCl_3_ resulted in a dark purple solid consisting of a mixture of boron 2,9,16-trifluorosubphthalocyanine chloride (F_3_SPc(C_3_)) and boron 2,9,17-trifluorosubphthalocyanine chloride (F_3_SPc(C_1_)) (Scheme 1). These two constitutional isomers were resolved by thin-layer chromatography (1:19 ethyl acetate/hexane) and purified by flash chromatography on silica gel using an eluent gradient of 0–5% ethyl acetate in hexane. Isolated yields of 4.7% and 7.4% were achieved for F_3_SPc(C_3_) and F_3_SPc(C_1_), respectively. Relatively low yields are indicative of the tedious and difficult chromatographic purification; however, the samples collected were highly pure. Purity was assessed and confirmed by HPLC using a diode-array detector (see Supplementary Materials).

**Figure 2:**
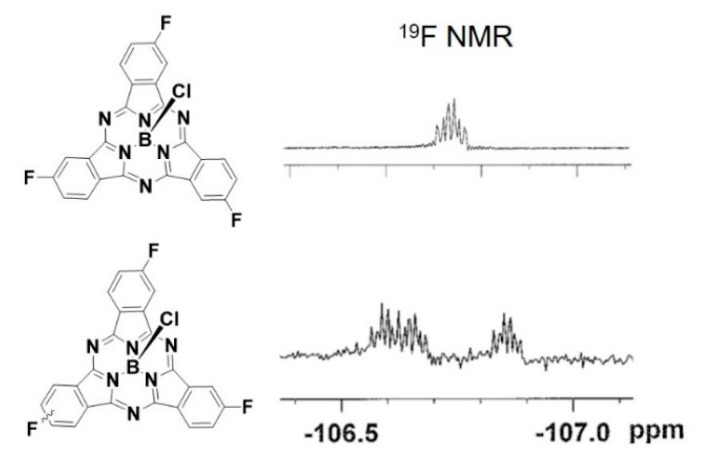
^19^F NMR of F_3_SPc(C_3_) (**upper**) and F_3_SPc(C_1_) (**lower**).

Identification of SPcs of constitutional isomers with very few ^1^H NMR signals can be difficult. Indeed, F_3_SPc(C_3_) and F_3_SPc(C_1_) display identical ^1^H NMR chemical shifts, with the only difference in spectra being the resolution of H–H and H–F coupling constants (see Supplementary Materials). One advantage of studying fluorinated SPcs with disparate symmetry is the ability to use ^19^F NMR to unequivocally identify isomers. The symmetric F_3_SPc(C_3_) shows one ^19^F NMR signal at δ −106.7 ppm (Figure 2). Breakage of the symmetry in F_3_SPc(C_1_) results in three separate signals at δ −106.6, −106.7, and −106.9 ppm. These β–F signals lie ~30 ppm downfield from those of SPcs with F-atoms in both α and β positions [20], in line with the trend that the extent of fluorination alters ^19^F chemical shifts further upfield.

### 2.2. Photophysical Properties of Subphthalocyanines

**Figure 3:**
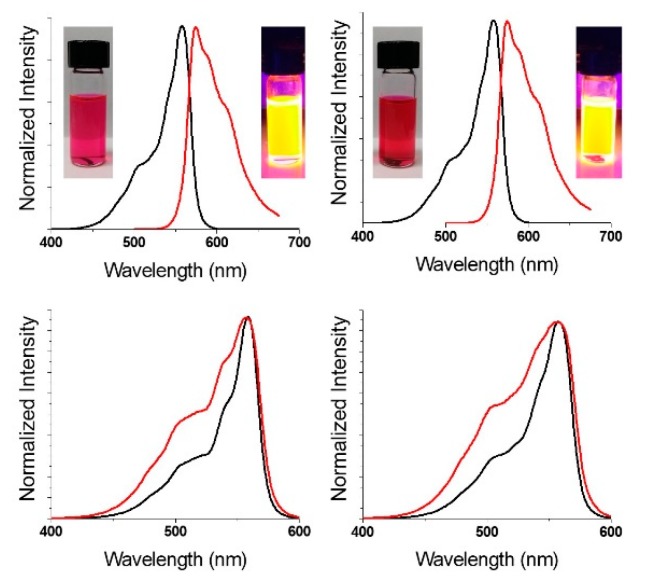
Normalized absorbance (**black**), emission (λ_ex_ = 325 nm (**red; upper**)) and excitation (λ_em_ = 625 nm (**red; lower**)) spectra of C_3_ (**left**) and C_1_ (**right**) F_3_-SPcs. Samples in THF solution.

Analogous to other pyrrolic macrocycles [28], SPcs have two major absorption features, the Soret band (2nd HOMO → LUMO; λ_max_ < 350 nm) and the Q-band (HOMO → LUMO; λ_max_ > 500 nm). The F_3_SPc(C_3_) and F_3_SPc(C_1_) exhibit identical Q-band absorptions at 557 nm, representing a ~15 nm hypsochromic shift from previously reported perfluorinated SPcs (Figure 3, black) [20]. Upon excitation into the Soret band (λ_ex_ = 325 nm), intense orange fluorescence is observed (λ_em_ = 575 nm) (Figure 3, upper, red). Excitation spectra (λ_em_ = 625 nm) match absorption spectra, as expected (Figure 3, lower). Quantum yields of fluorescence (Φ_f_) were determined by the relative method, using boron subphthalocyanine chloride as a standard (Φ_f_ = 0.29). Quantum yields were found to be identical for the constitutional isomers (Φ_f_ = 0.27–0.28) within the error of the measurement. Furthermore, these values are comparable to other fluorinated SPcs, providing sufficient brightness for fluorescence imaging studies, discussed below.

### 2.3. Epifluorescence Microscopy of Subphthalocyanines in MDA-MB-231 Breast Tumor Cells

**Figure 4:**
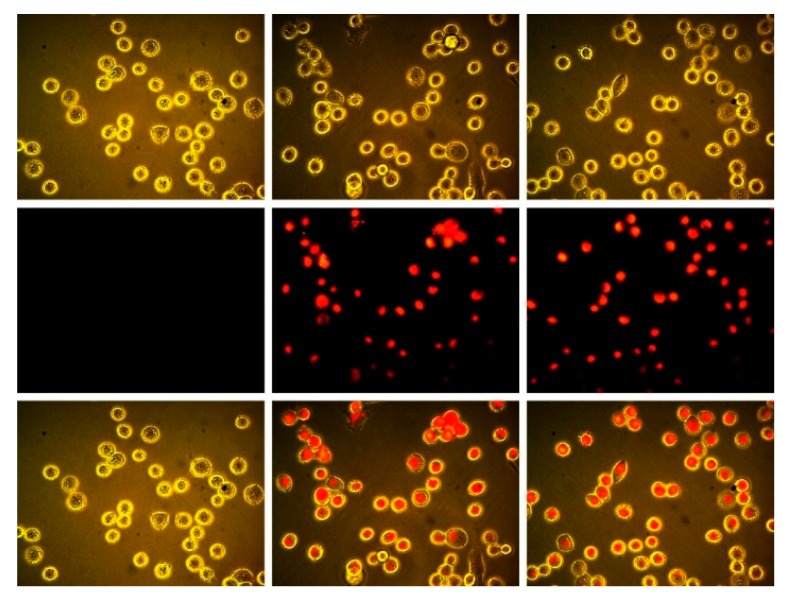
Brightfield (**upper**), epifluorescence ((**middle**) λ_ex_ = BP 528–553 nm, λ_em_ = BP 578–633 nm), and (**lower**) overlay images of MDA-MB 231 breast tumor cells. DMSO control (**left**), treated with 50 μM F_3_SPc(C_3_) (**center**), and 50 μM F_3_SPc(C_1_) (**right**) for 15 min (400×).

As discussed above, we had previously reported that the inclusion of F-atoms at the periphery of boron subpthalocyanine chloride macrocycles led to enchanced intracellular fluorescence from the SPcs in MDA-MB-231 cells. In these examples, each isoindoline sub-unit was perfluorinated, yielding SPcs with either 4, 8, or 12 F-atoms at the periphery. To further test this platform, trifluorosubphtlaocyanines reported here were tested in MDA-MB-231 cells. The difference reported herein is that SPcs have fewer F-atoms overall (3 vs. 4–12) and are situated only in the β positions, as opposed to both α and β positions. Cells were plated and allowed to grow to 50% confluency before being treated with 50 μM F_3_SPc(C_3_) (Figure 4, center) and 50 μM F_3_SPc(C_1_) (Figure 4, right), using 1% DMSO as a solubilizing vehicle. Control cells were treated with the 1% DMSO vehicle alone (Figure 4, left).

Treated cells showed marked intracellular fluorescence compared to the vehicle control. Therefore, even as few as three F-atoms at the SPc periphery can lead to enhanced cellular uptake, and possession of F-atoms in the α position on the isoindoline is not a necessary requirement.

## 3. Materials and Methods

### 3.1. General Considerations

Reagents and chemicals, including silica gel (60 Å, 230–400 mesh), were purchased from VWR (Radnor, PA, USA) and used without further purification unless otherwise noted. Nuclear magnetic resonance (NMR) spectra were recorded on an Avance III (400 MHz, Bruker, Billerica, MA, USA) spectrophotometer. ^19^F NMR spectra were recorded using trifluoroacetic acid as a standard (δ = −76.55 ppm). Mass spectrometry was performed on an LC/MS 6545 Q-TOF (Agilent, Santa Clara, CA, USA) in APCI mode. Purity analysis by HPLC was performed on an Agilent 1100 system with a diode array detector and C8 ZORBAX Eclipse Plus column (Agilent). Absorption data was collected on a Cary-100 UV-vis spectrophotometer (Agilent) in double-beam mode using 1-cm path quartz cuvettes. Corrected fluorescence spectra were collected on a Fluorolog 3 fluorometer (Horiba Jobin-Yvon, Kyoto, Japan) equipped with an R928 PMT (Hamamatsu, Bridgewater Township, NJ, USA). Solutions were prepared such that absorption remained below 0.1 AU to prevent reabsorption and self-quenching.

### 3.2. Synthesis

*Boron trifluorosubphthalocyanine chloride*. To a 25 mL round-bottomed flask was added 4-fluorophthalonitrile (0.134 g, 0.92 mmol). The flask was purged with N_2_, *p*-xylene (4 mL) was added and the flask was heated to 140 °C. A solution of BCl_3_ (1 M in *p*-xylene, 0.75 mL, 0.75 mmol) was added, resulting in a yellow solution. After approximately five minutes, the solution changed color to dark purple. The reaction was stirred for 40 min, after which the solvent was reduced in vacuo. The resulting dark purple solid was dissolved in dichloromethane and filtered through a plug of silica gel. The resulting sample was purified by flash chromatography on silica gel (0–5% ethyl acetate gradient in hexane).

*F_3_SPc(C_3_)—Boron 2,9,16-trifluorosubphthalocyanine chloride*. First elution, purple solid (0.007 g, 0.015 mmol, 4.7% yield). ^1^H NMR (400 MHz, CDCl_3_) δ (ppm): 8.87 (dd, 3H, *J*_H–H(ortho)_ = 8.7 Hz, *J*_H–F(meta)_ = 4.7 Hz), 8.53 (dd, 3H, *J*_H–F(ortho)_ = 8.2 Hz, *J*_H–H(meta)_ = 2.2 Hz), 7.69 (ddd, 3H, *J*_H–H(ortho)_ = *J*_H–F(ortho)_ = 8.7 Hz, *J*_H–H(meta)_ = 2.3 Hz). ^19^F NMR (376 MHz, CDCl_3_) δ (ppm): −106.74 (m). HR-LCMS APCI: calcd. for (C_24_H_10_BClF_3_N_6_) [M + H]^+^, 485.0701; found, 485.0702.

*F_3_SPc(C_1_)—Boron 2,9,17-trifluorosubphthalocyanine chloride*. Second elution, purple solid (0.011 g, 0.025 mmol, 7.4% yield). ^1^H NMR (400 MHz, CDCl_3_) δ (ppm): 8.87 (m, 3H), 8.52 (m, 3H), 7.68 (m, 3H). ^19^F NMR (376 MHz, CDCl_3_) δ (ppm): −106.60 (m), −106.66 (m), −106.86 (m). HR-LCMS APCI: calcd. for (C_24_H_10_BClF_3_N_6_) [M + H]^+^, 485.0701; found, 485.0704.

### 3.3. Fluorescence Quantum Yield Determination

Fluorescence quantum yields were determined by the relative method [29], and analysis with Equation (1):

(1) $$\Phi_{x}=\Phi_{r}\cdot \left(\frac{A_{r}\cdot F_{x}\cdot n_{x}{}^{2}}{A_{x}\cdot F_{r}\cdot n_{r}{}^{2}}\right)$$

where *r* and *x* denote the standard and unknown, respectively, *A* is the absorption intensity at the excitation wavelength, *F* is the integrated fluorescence intensity and *n* is the refractive index of the solvent. Cross-calibration determined less than 10% error for this method and instrumentation.

### 3.4. Cell Culture and Fluorescence Imaging

Cell culture reagents were obtained from Hyclone (GE, Boston, MA, USA) unless otherwise noted. MDA-MB-231 breast tumor cells were obtained from the American Type Culture Collection and maintained in Dulbecco′s Modified Eagle Medium (DMEM) supplemented with 10% fetal bovine serum. For imaging assays, cells were plated in 12-well plates (Corning, Corning, NY, USA) and allowed to grow to ~50% confluency before being treated with solutions of SPcs in media (1% dimethylsulfoxide). Images were collected with a VWR Inverted Fluorescence Microscope, Moticam 5.0 (Motic, Richmond, BC, Canada), and TRITC filter set (λ_ex_ = 540 nm ± 12 nm, λ_em_ = 605 nm ± 27 nm). 

## Supplementary Materials

The following are available online. HR APCI-MS, ^1^H NMR, ^19^F NMR, and HPLC chromatograms with DAD purity reports.
