# Supplementary material for: Synthesis of the C3 and C1 Constitutional Isomers of Trifluorosubphthalocyanine and Their Fluorescence within MDA-MB-231 Breast Tumor Cells
Source: Molecules. 2019 Oct 24;24(21):3832. doi: 10.3390/molecules24213832 (PMC6864546; doi:10.3390/molecules24213832)
Supplement: Supplementary file 1 [file molecules-24-03832-s001.pdf]

Supporting Information for:

# Synthesis of the $C_3$ and $C_1$ Constitutional Isomers of Trifluorosubphthalocyanine and their Fluorescence within MDA-MB-231 Breast Tumor Cells

**Rosemarie L. Calandrino, Katherine J. McAuliffe †, Lauren E. Dolmage and Evan R. Trivedi ***

Department of Chemistry, Oakland University, Rochester, MI 48309, USA; rcalandrino@oakland.edu (R.L.C.); katherine.mcauliffe@duke.edu (K.J.M.); laurendolmage@oakland.edu (L.E.D.)

† Present Addresses: Department of Chemistry, Duke University, Durham, NC 27708, USA.

* Correspondence: trivedi@oakland.edu; Tel.: +01-248-370-2147; Fax: +01-248-370-2321

Table of Contents:

**Figure S1** – HR APCI-MS **$F_3$SPc($C_3$)**……S2
**Figure S2** – HR APCI-MS **$F_3$SPc($C_1$)**……S2
**Figure S3** – Aromatic $^1$H NMR **$F_3$SPc($C_3$)**……S3
**Figure S4** – Aromatic $^1$H NMR **$F_3$SPc($C_1$)**……S3
**Figure S5** – $^{19}$F NMR **$F_3$SPc($C_3$)**……S4
**Figure S6** – $^{19}$F NMR **$F_3$SPc($C_1$)**……S4
**Figure S7** – HPLC purity report **$F_3$SPc($C_3$)**……S5
**Figure S8** – HPLC purity report for **$F_3$SPc($C_1$)**……S6

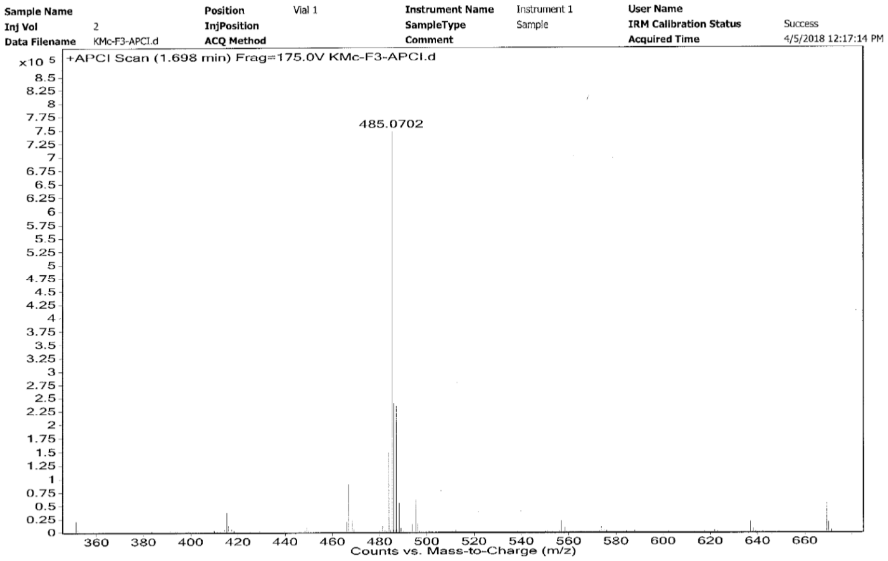


**Figure S1.** – HR APCI-MS $F_3SPc(C_3)$.

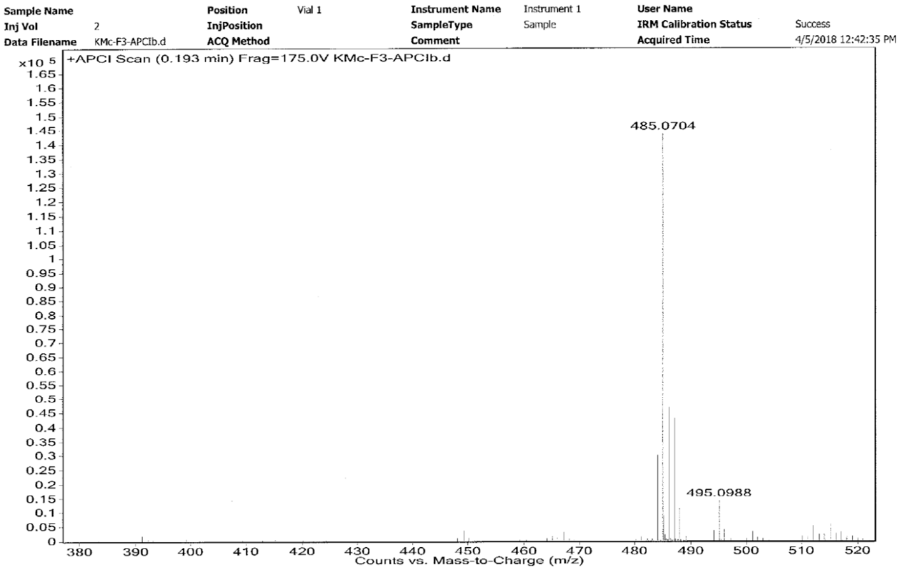


**Figure S2.** – HR APCI-MS $F_3SPc(C_1)$.

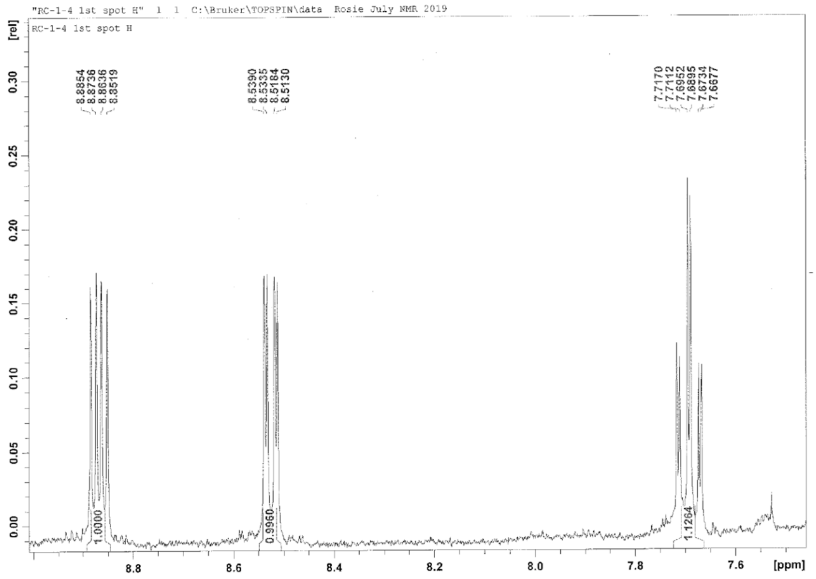


**Figure S3.** – Aromatic $^{1}$H NMR $F_3SPc(C_3)$.

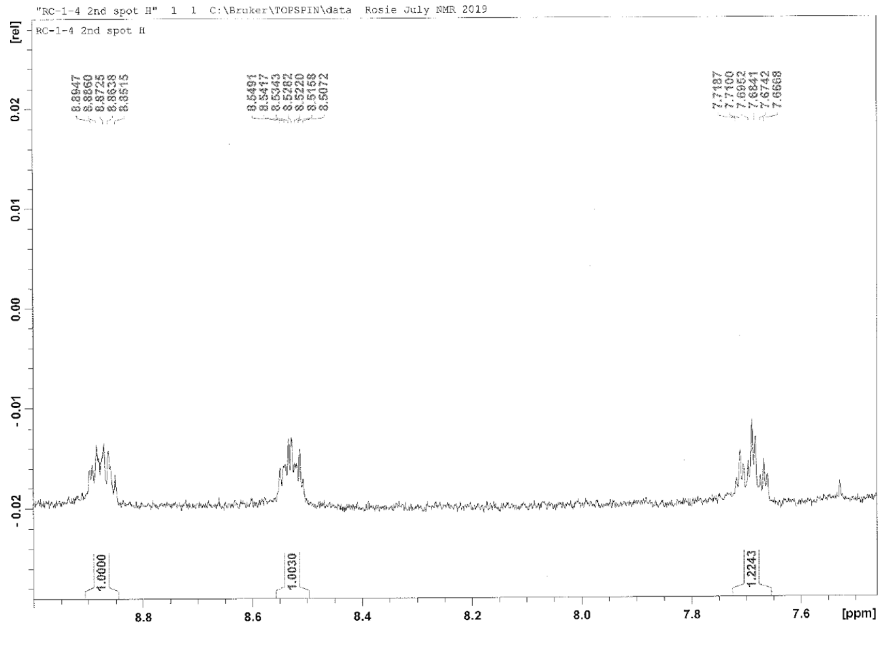


**Figure S4.** – Aromatic $^{1}$H NMR $F_3SPc(C_1)$.

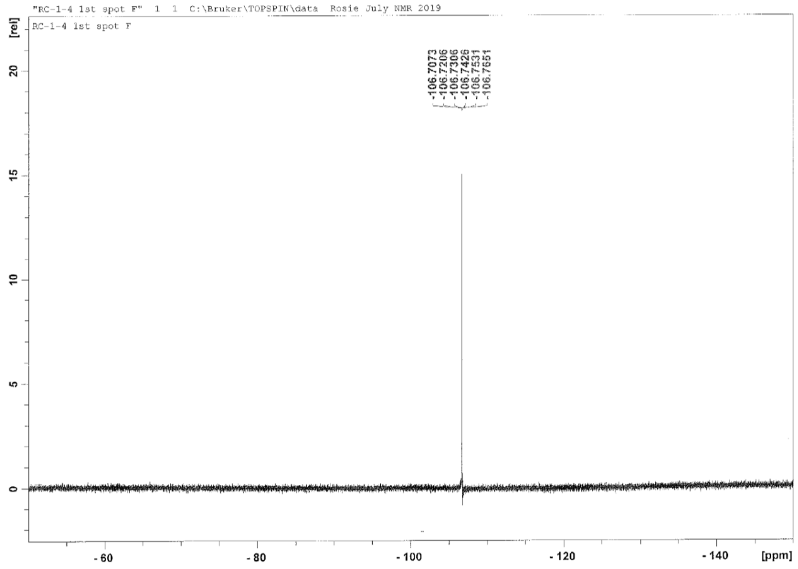


**Figure S5.** – $^{19}F$ NMR $F_3SPc(C_3)$.

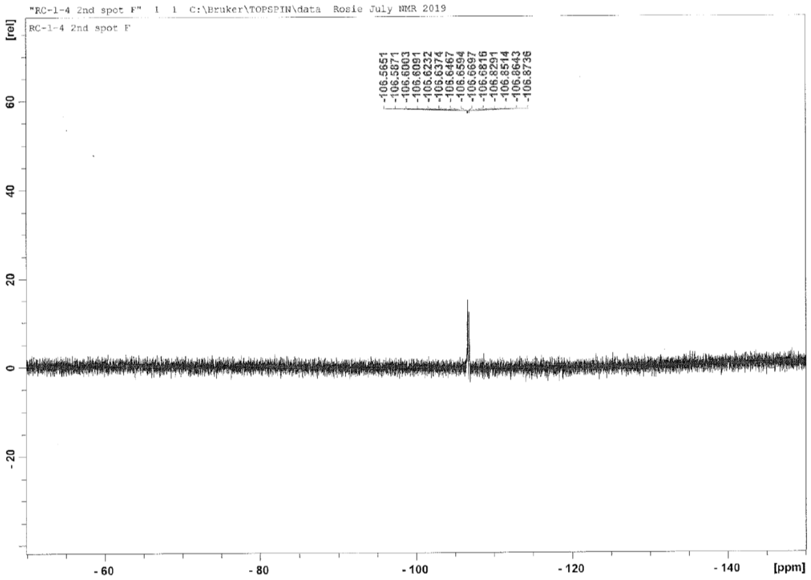


**Figure S6.** – $^{19}F$ NMR $F_3SPc(C_1)$.

Purity results peak 1 at 11.025 min.

Signal DAD1 B, Sig=550,16 Ref=off (RC\RC-1-4-2NDCOL02.D)

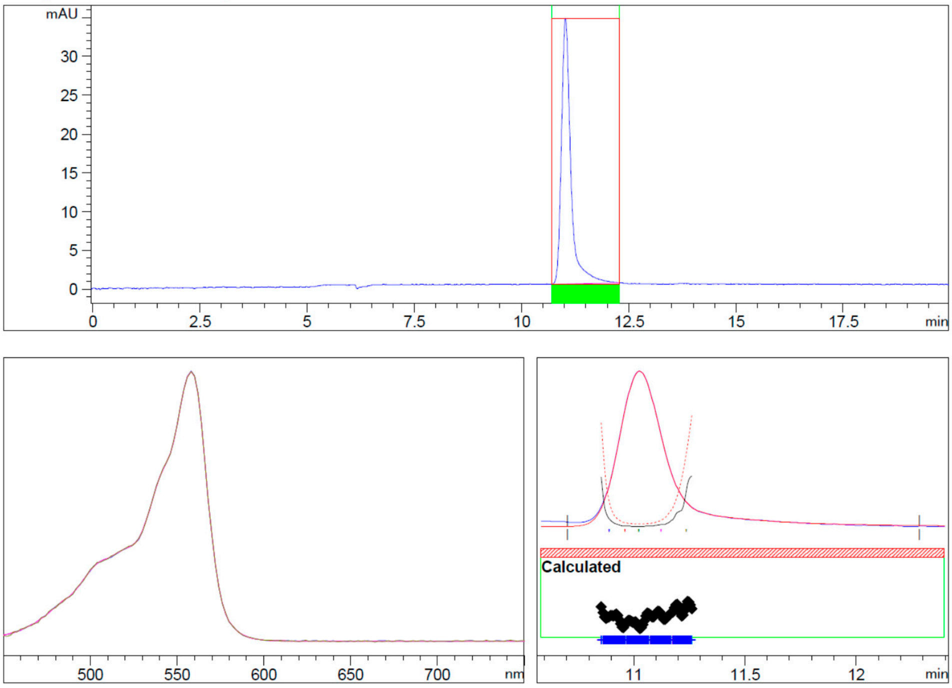


-> The purity factor is within the calculated threshold limit. <-

Purity factor : 999.985 (62 of 62 spectra are within the calculated threshold limit.)
Threshold : 999.961 (Calculated with 62 of 62 spectra)
Reference : Peak start and end spectra (integrated) (10.703 / 12.283)
Spectra : 5 (Selection automatic, 5)
Noise Threshol: 0.018 (12 spectra, St.Dev 0.009 + 3 * 0.003)

*** End of Report ***

**Figure S7.** – HPLC purity report **$F_3SPc(C_3)$.**

Purity results peak 1 at 10.708 min.

Signal DAD1 B, Sig=550,16 Ref=off (RC\RC-1-4-2NDCOL03.D)

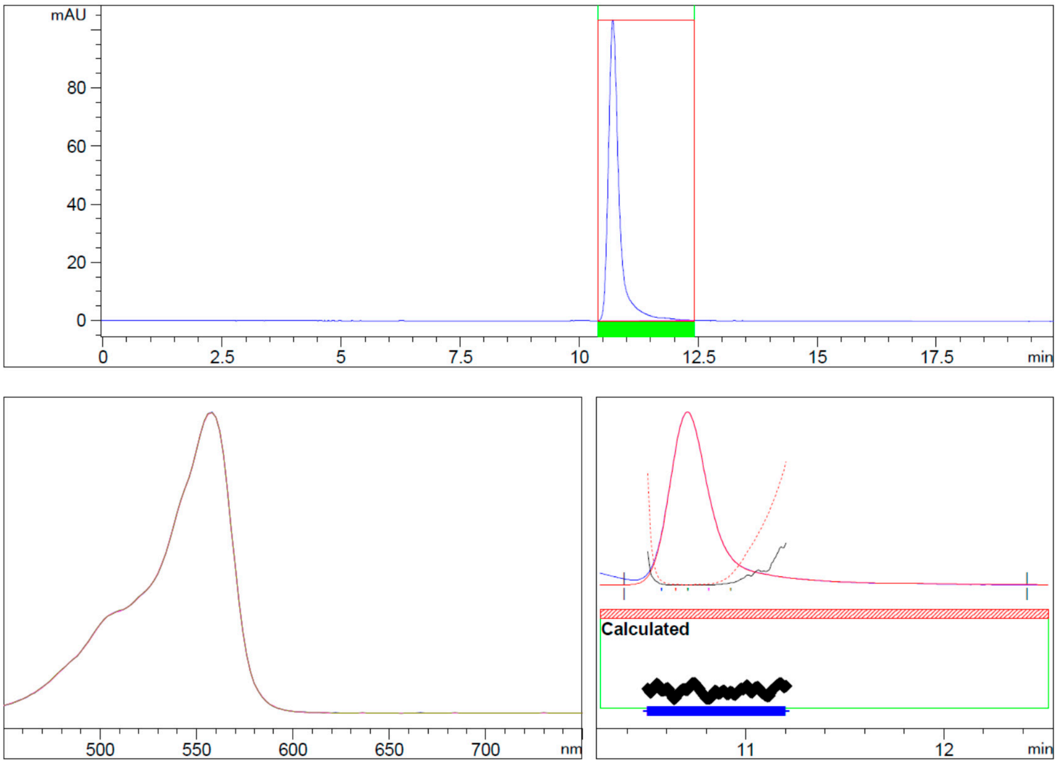


-> The purity factor is within the calculated threshold limit. <-

Purity factor : 999.984 (106 of 106 spectra are within the calculated threshold limit.)
Threshold : 999.936 (Calculated with 106 of 106 spectra)
Reference : Peak start and end spectra (integrated) (10.384 / 12.418)
Spectra : 5 (Selection automatic, 5)
Noise Threshol: 0.024 (12 spectra, St.Dev 0.0111 + 3 * 0.0043)

*** End of Report ***

**Figure S8.** – HPLC purity report for **$F_3$SPc($C_1$).**
